# Mandibular angle osteotomy in Asian patients: A narrative review on techniques, emerging technologies, and personalized surgical strategies

**DOI:** 10.1016/j.jpra.2026.06.008

**Published:** 2026-06-22

**Authors:** Zhiyang Xie, Xueshan Bai, Ziying Guo, Liya Yang, Li Teng

**Affiliations:** Department of Craniomaxillofacial Surgery, Plastic Surgery Hospital, Chinese Academy of Medical Sciences and Peking Union Medical College, Beijing, 100144, China

**Keywords:** Surgical strategy, Mandibular angle osteotomy, Prominent mandibular angle, Facial contouring surgery

## Abstract

**Background:**

Mandibular angle osteotomy (MAO) is widely performed in East Asian patients seeking lower-face contouring. Although numerous osteotomy designs and adjunctive technologies have been described, practical guidance for selecting an appropriate technique according to mandibular morphology, surgical endpoints, and surgeon experience remains limited.

**Methods:**

A narrative review was conducted using a systematic literature search of PubMed and Embase up to December 30, 2025. Studies related to MAO techniques, anatomical classification, aesthetic assessment, adjunctive procedures, emerging technologies, complications, and long-term remodeling were reviewed.

**Results:**

MAO techniques differ mainly in the starting point, endpoint, and trajectory of the osteotomy line. Long curved osteotomy remains a commonly used approach for prominent mandibular angles, whereas ABC osteotomy, U-shaped osteotomy, V-line osteotomy, mandibular outer cortex reduction, mandibular body osteotomy, genioplasty, and partial masseter muscle resection may be selected according to specific skeletal and soft-tissue features. Surgical endpoints should include not only skeletal reduction but also frontal width, lateral contour, mandibular border smoothness, symmetry, soft-tissue support, neurosensory safety, patient satisfaction, and long-term remodeling. Digital planning, osteotomy templates, navigation, and endoscopic assistance may improve accuracy and safety, especially in anatomically complex cases.

**Conclusion:**

MAO should be planned as an individualized lower-face contouring procedure rather than a uniform reduction technique. A morphology-based decision-making framework may help clarify indications, optimize surgical endpoints, reduce complications, and improve long-term aesthetic outcomes. Future studies should emphasize standardized outcome measures, patient-reported outcomes, and long-term three-dimensional assessment.

**Level of evidence:**

V

## Introduction

Prominent mandibular angle (PMA) was first described by Baek et al. in 1989,^1^ after which mandibular angle osteotomy (MAO) gradually became a widely adopted aesthetic procedure, particularly among East Asian women seeking a slimmer and more harmonious lower facial contour. The intraoral approach has largely replaced the extraoral approach owing to its advantages of inconspicuous scarring and reduced risk of facial nerve injury.^2^, ^3^, ^4^ With the growing demand for facial contouring surgery, MAO has become one of the most effective techniques for reshaping the lower face.^5^^,^^6^

Several modified MAO techniques have been developed to accommodate variations in mandibular anatomy, primarily by adjusting the osteotomy design, including the start point, end point, and trajectory. Representative procedures include the long curved osteotomy (LC osteotomy),^3^ en bloc mandibular angle–body–chin curved ostectomy (ABC osteotomy),^7^ en bloc U-shaped osteotomy of the mandible and chin (U osteotomy),^8^ and mandibular V-line ostectomy (V osteotomy).^9^ In addition, for patients seeking improvement in the frontal view, mandibular outer cortex reduction (by grinding or splitting)^7^^,^^4^ and/or partial masseter muscle resection (PMMR)^10^, ^11^, ^12^ may be considered. Advances in digital technologies—including virtual surgical planning (VSP),^13^^,^^14^ digital osteotomy templates (DOT),^15^, ^16^, ^17^, ^18^, ^19^, ^20^, ^21^, ^22^ and dynamic real-time navigation—have also emerged as adjuncts for MAO. Collectively, these technical refinements and technological innovations have contributed to improved outcomes and reduced complication rates.

Despite the increasing use of MAO and related lower-face contouring procedures, the available literature remains fragmented, with most studies focusing on individual techniques rather than integrated surgical decision-making. For international readers, including surgeons practicing in Europe, a practical framework is needed to clarify how Asian mandibular morphology, patient expectations, technical feasibility, and surgeon experience should be balanced when selecting an osteotomy design. Therefore, this narrative review aims to summarize current MAO techniques, define clinically relevant surgical endpoints, discuss key controversies, and propose a morphology-based decision-making framework for individualized lower-face contouring.

## Methods

This narrative review was conducted using a systematic literature search of PubMed and Embase up to December 30, 2025. The search terms included “mandibular angle osteotomy,” “mandibular angle reduction,” and “prominent mandibular angle.” Studies were considered eligible if they reported surgical techniques, anatomical classification, aesthetic assessment, digital planning, intraoperative guidance, postoperative outcomes, complications, or long-term remodeling related to MAO or lower-face contouring. Articles unrelated to mandibular contouring, studies focusing exclusively on oncologic mandibular reconstruction, and non-clinically relevant technical reports were excluded. The included literature was synthesized narratively to develop a practical decision-making framework rather than to perform a quantitative meta-analysis.

## Results and discussion

**Figure 1:**
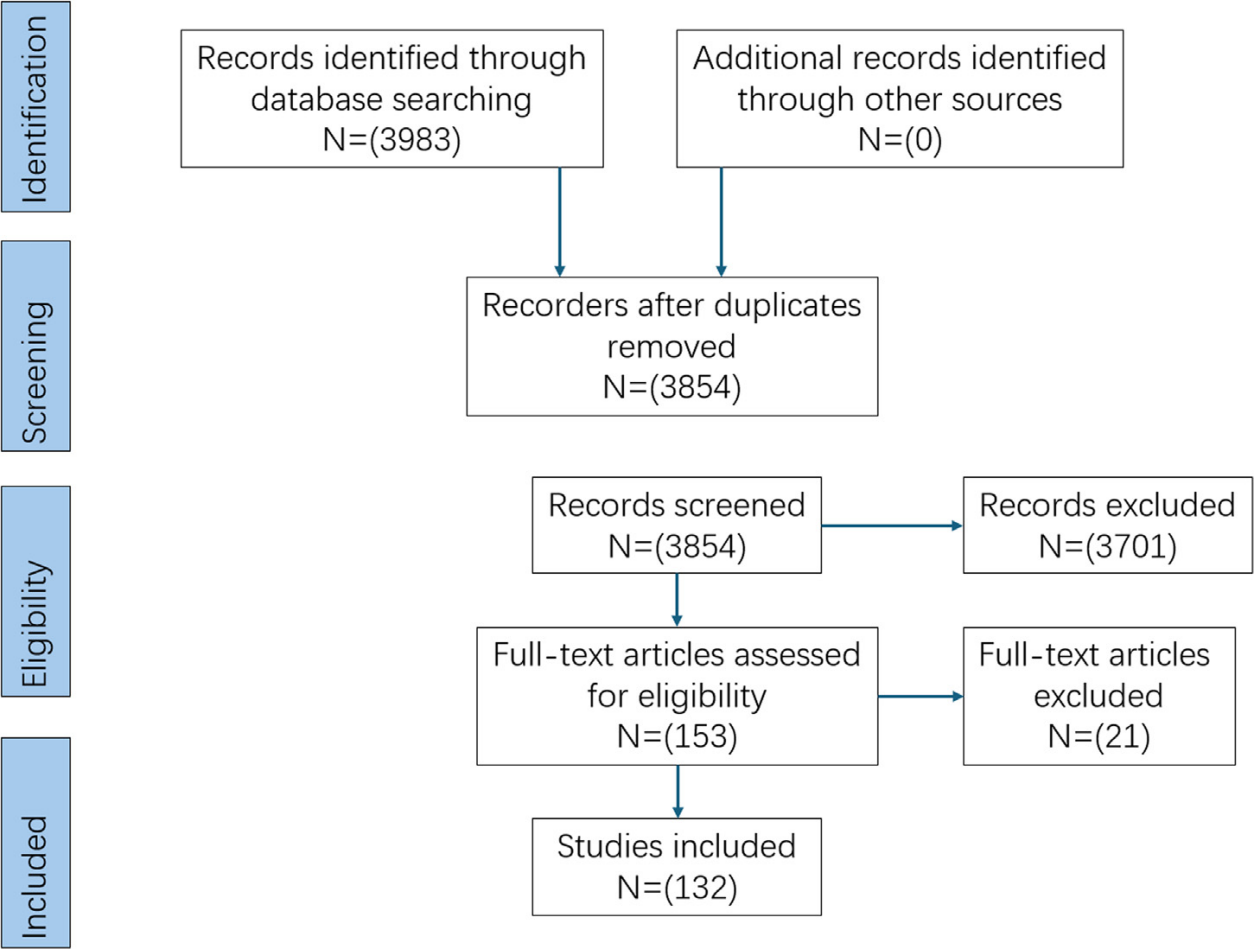
Flow diagram summarizing the search results.

**Figure 2:**
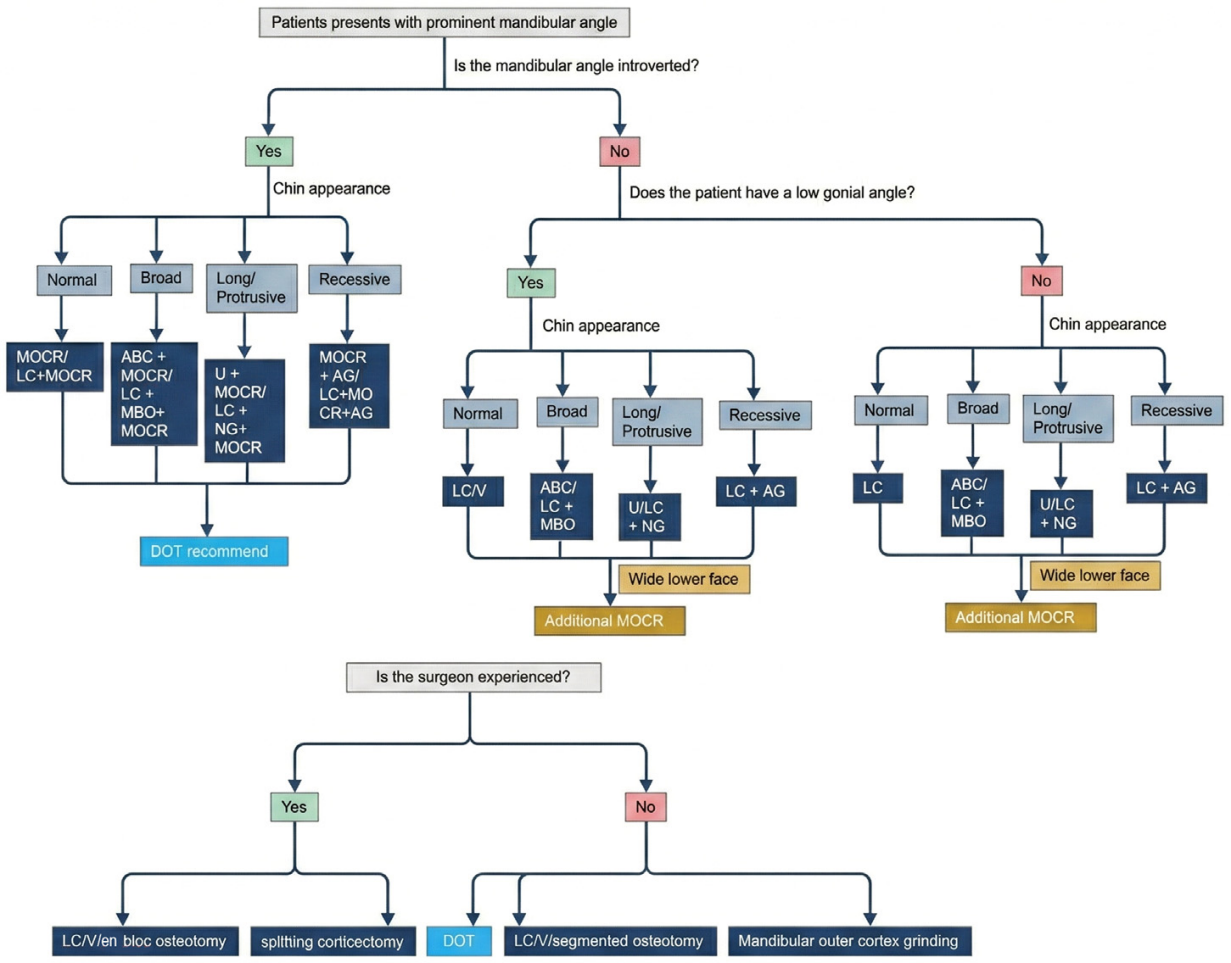
Surgical decision-making tree for mandibular angle osteotomy. Osteotomy options include long curved osteotomy (LC), en bloc mandibular angle–body–chin curved ostectomy (ABC), en bloc U-shaped osteotomy of the mandible and chin (U), and mandibular V-line ostectomy (V), with adjunctive procedures including narrowing genioplasty (NG), advancing genioplasty (AG), mandibular outer cortex reduction (MOCR), mandibular body osteotomy (MBO), and the use of digital osteotomy templates (DOT).

Clinical studies included in this review were identified according to the process illustrated in Figure 1. The surgical decision-making tree is presented in Figure 2.

### Classification of prominent mandibular angle

**Figure 3:**
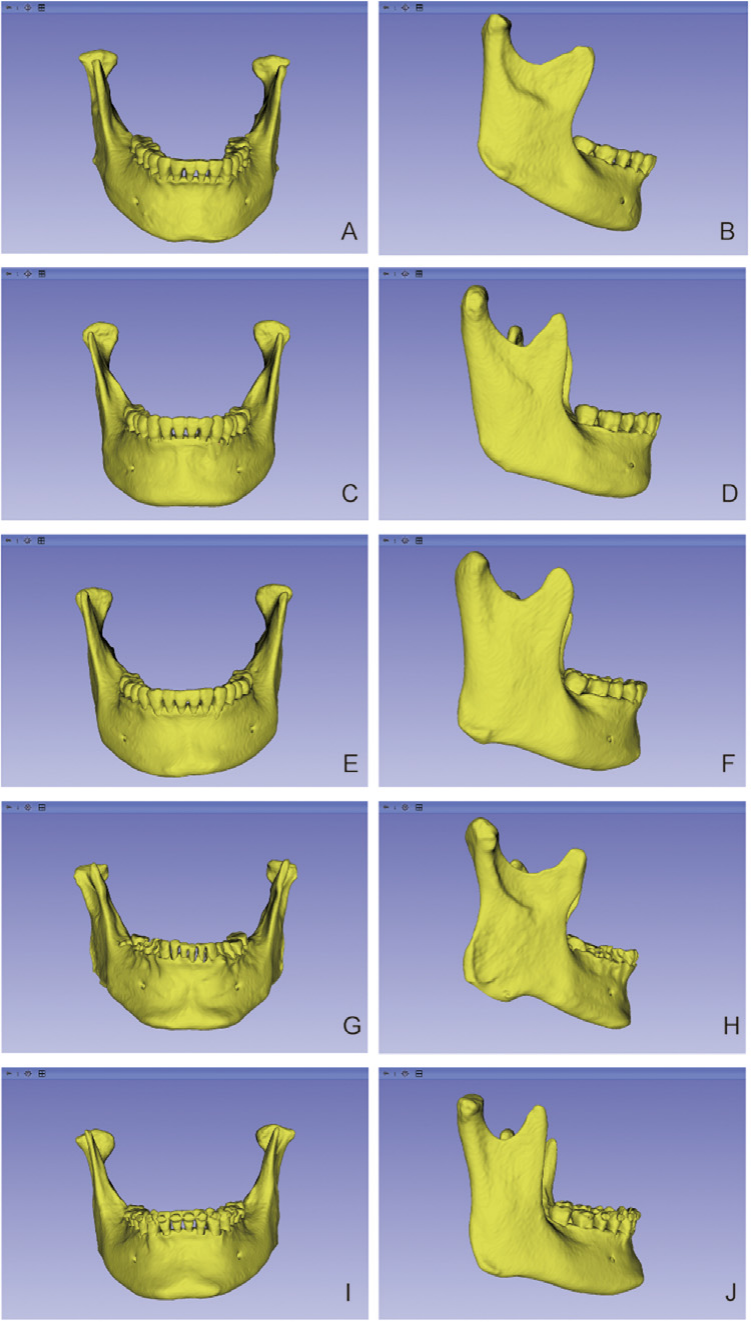
Classification of prominent mandibular angle. (A, B) Extroverted type, frontal and lateral views; (C, D) Introverted type, frontal and lateral views; (E, F) Low gonial angle type, frontal and lateral views; (H, I) Extroverted + low gonial angle type, frontal and lateral views; (J, K) Introverted + low gonial angle type, frontal and lateral views.

Currently, there is no unified classification standard for prominent mandibular angle.^23^, ^24^, ^25^, ^26^ In this study, we adopted the classification standard proposed by Xie et al.^26^ PMA was categorized into four types based on this criterion: (1) Extroverted: Mandibular angle extends beyond the sagittal plane; (2) Introverted: Mandibular angle lies within the sagittal plane; (3) Low Gonial Angle: Mandibular angle projects posteriorly in the coronal plane with a lower ∠Co–Go–Me and MP-HP angle; (4) Mixed: A combination of extroverted or introverted morphology with a low gonial angle. Three-dimensional reconstructions of the four types in frontal and lateral views are shown in Figure 3.

### Morphological assessment and surgical endpoints of lower facial contour

As a three-dimensional structure, the mandible requires a comprehensive assessment from frontal, oblique and lateral views, taking into account the height, morphology, and divergence of the mandibular angle, body, and chin in patients with normal occlusion.^25^

#### Lateral view

The morphology and position of mandibular angle play a key role in the lateral view. A well-defined mandibular angle is characterized by an appropriate condylion–gonion–menton angle (∠Co–Go–Me) and mandibular plane–horizontal plane (MP–HP) angle, which differs between males and females. Based on measurements of attractive Chinese females, a ∠Co–Go–Me between 115° and 128° and a MP–HP angle between 22° to 28° are generally considered aesthetically favorable among Chinese females.^20^^,^^27^ The earlobe-to-gonial angle distance, typically about 2 cm in an aesthetically pleasing lateral profile.^24^ In males, the mandibular angle generally ranges between 120° and 130°, contributing to a masculine contour.^6^ Mao et al.^28^ proposed a Fisher linear discriminant function to identify attractive female facial profiles, expressed as follows: *Y* = −0.1516*X*_1_(Co–Go) + 0.128*X*_2_(Go–Me) + 0.04936*X*_3_(Co–Go–Me) + 0.0218*X*_4_(FH–MP).

#### Oblique view

The mandibular body acts as the transitional segment between the mandibular angle and the chin. An aesthetically ideal mandibular body features a smooth, continuous, and harmonious curve from the gonial angle to the menton, with an uninterrupted and subtle inferior border.^29^

#### Frontal view

The lower facial width and chin appearance significantly influence the frontal facial view. With respect to lower facial width, aesthetic evaluation should not rely on the absolute gonion-to-gonion (Go–Go) distance alone, but rather on its proportion relative to midfacial width, commonly expressed as the Go–Go to zygoma point–to–zygoma point (Zy–Zy) ratio. In a measurement-based aesthetic study by Wu et al.,^30^ visual analog scale (VAS) evaluation demonstrated that a Go–Go/Zy–Zy ratio of approximately 75% was perceived as the most aesthetically pleasing facial taperness. Ideal chin proportions follow the equal-thirds facial canon, with a 1:2 ratio within the lower facial third and a projection that aligns with the Ricketts esthetic line.^31^ Nevertheless, surgical planning should always consider patient preferences, overall facial harmony, and the relationship between hard and soft tissues.

These aesthetic parameters provide a quantitative framework to guide osteotomy design; however, their application should remain flexible and individualized rather than prescriptive.

### Surgical endpoints and outcome assessment

In the context of mandibular angle osteotomy, surgical endpoints should not be limited to the amount of bone resection. Instead, successful lower-face contouring should be evaluated using a multidimensional framework that includes skeletal contour, frontal facial width, lateral mandibular angle definition, smoothness of the mandibular inferior border, facial symmetry, soft-tissue response, neurosensory safety, occlusal stability, patient satisfaction, and long-term skeletal remodeling. For Asian patients seeking lower-face narrowing, the desired endpoint is usually a smooth and harmonious mandibular contour rather than maximal angular reduction. Therefore, preoperative planning should define individualized endpoints according to mandibular morphology, chin proportion, masseter volume, soft-tissue thickness, and patient expectations.

### General procedure of MAO

MAO via an intraoral approach generally involved preoperative CT-guided osteotomy planning, intraoral incision, subperiosteal dissection to full exposure of mandibular body, angle, ramus and metal nerve, osteotomy along the predesigned line, contour refinement, and wound closure after symmetry confirmation. Optional procedures such as mandibular outer cortex reduction (MOCR), partial masseter muscle resection (PMMR), or genioplasty are considered based on the patient's characteristics.^6^

### Mandibular angle full thickness osteotomy line design and variations

**Figure 4:**
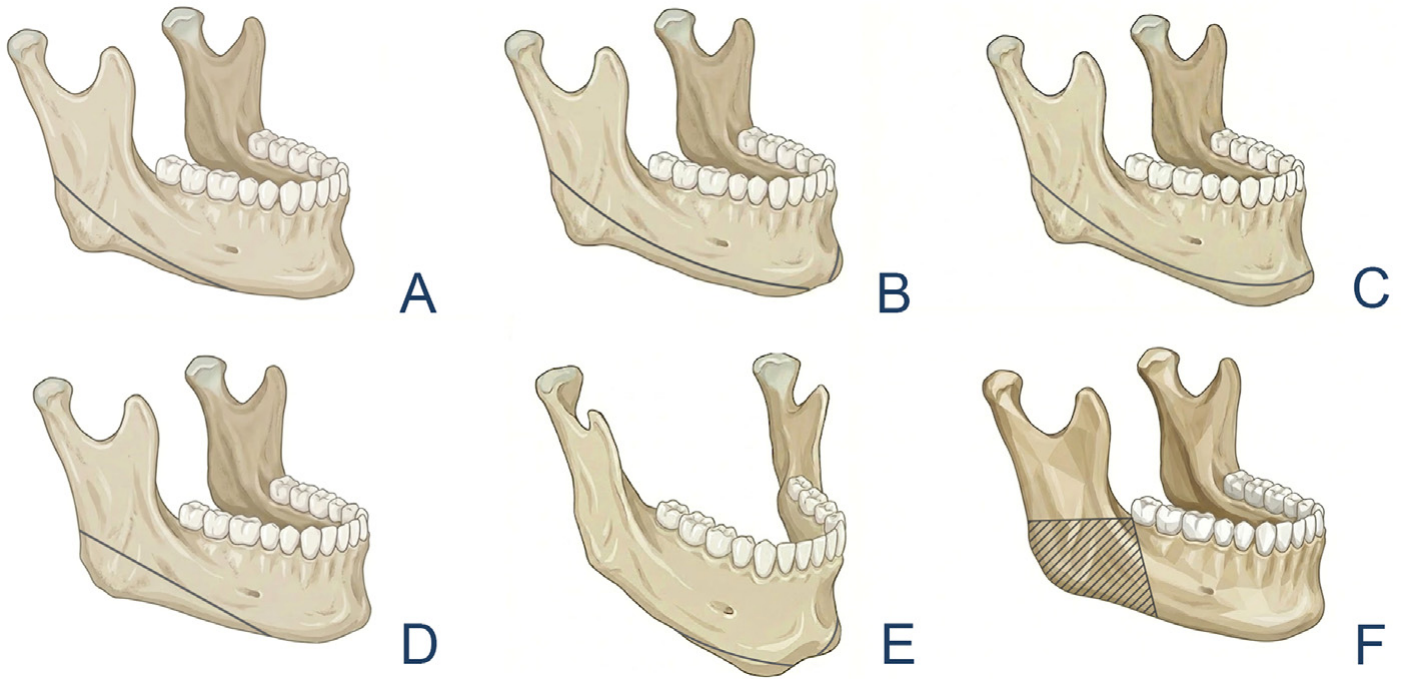
Schematic illustration of osteotomy line designs for different mandibular contouring techniques. (A) Long curved osteotomy; (B) En bloc mandibular angle–body–chin curved ostectomy; (C) En bloc U-shaped osteotomy of the mandible and chin; (D) Mandibular V-line ostectomy; (E) Mandibular body osteotomy; (F) Mandibular outer cortex reduction.

The osteotomy line should be designed with careful consideration of the mandibular anatomy. The occlusal plane serves as the reference for determining the starting point of the osteotomy, while the endpoint should be tailored according to each patient’s anatomical features and aesthetic requirements. Regardless of the starting point, endpoint, or direction of the osteotomy line, a minimum distance of 3–5 mm must be preserved between the osteotomy trajectory and the inferior alveolar neurovascular bundle.^7^^,^^8^ Key aspects of osteotomy line design are outlined in Table 1. Schematic illustrations of the surgical procedures are shown in Figure 4. The surgical details were discussed below.

**Table 1:** Design, indications, advantages, and limitations of different mandibular angle osteotomy techniques.

Surgical technique	Osteotomy design	Main indications	Advantages	Limitations
Long curved mandibular angle osteotomy.^3^	The curved osteotomy line extends from the occlusal plane of the mandibular ramus to a point below the mental foramen.	Patient complains of a prominent mandibular angle as the sole concern.	It is broadly applicable to most patients and is characterized by its simplicity and ease of execution.	It’s not suitable for patients with mandibular body and chin deformities.
En-bloc mandibular angle-body-chin curved ostectomy.^7^	The curved osteotomy line extends from the occlusal plane of the mandibular ramus to the inferior mandibular border.	Patient complains of a prominent mandibular angle with a broad chin.	It eliminates the need for multistage procedures.	It is associated with a relatively higher incidence of temporary lower-lip numbness and facial sagging.
En-bloc mandibular U-shaped ostectomy.^8^^,^^35^	The curved osteotomy removes the entire inferior mandibular border as a continuous U-shaped segment.	Patient complains of a prominent mandibular angle with a wide and long chin, especially for patients with a low-positioned inferior alveolar nerve canal.	It eliminates the need for multistage procedures.	It is associated with a relatively higher incidence of temporary lower-lip numbness and facial sagging, especially in the submental-cervical region.
Mandibular V-line osteotomy.^9^	The osteotomy lines on both sides are symmetrically designed to create a configuration resembling the letter “V.”	It's suitable for patients with a low gonial angle or those seeking an extremely slender oval facial contour.	It is technically simpler than the curved osteotomy.	It is not suitable for patients with a high-positioned mandibular angle or an excessively short mandibular ramus.

#### Long curved mandibular angle osteotomy

LC osteotomy was first reported by Gui et al. in 2005,^3^ where it was initially described as the one-stage curved osteotomy to avoid the formation of second angle. Since then, it has served as the foundational technique for curved MAO. Owing to its broad applicability to most patients, it has become the mainstream and standard approach for MAO. The osteotomy line was individually designed to follow a smooth, continuous curvature and to terminate below the mental foramen.^32^^,^^33^ It should be noted that this technique is not suitable for patients with mandibular body and chin deformities, for whom genioplasty or alternative procedures are required.

#### En-bloc mandibular angle-body-chin curved ostectomy

To address PMA accompanied by a broad chin, Zhang et al.^7^ extend the osteotomy line from occlusal plane of the mandibular ramus to the inferior mandibular border below the lateral incisor. ABC osteotomy eliminates the need for multistage procedures, thereby minimizing surgical trauma and reducing operative time. However, it is important to note that en-bloc osteotomy is associated with a relatively higher incidence of temporary lower-lip numbness, primarily due to extensive soft-tissue dissection and intraoperative traction. To reduce the risk of nerve injury, the oscillating saw is used only until the osteotomy line reaches a position inferior to the mental nerve, after which a reciprocating saw is employed to complete the osteotomy safely. In addition, the removal of the en-bloc bone fragment can be technically challenging, particularly for less experienced surgeons.

#### En-bloc mandibular U-shaped osteotomy

U osteotomy was first reported by Dr. Jin’s team in 2019^8^ and was designed for patients diagnosed with PMA accompanied by a long or wide chin. They developed a single-stage intraoral procedure that removes the entire inferior mandibular border as a continuous U-shaped segment, allowing simultaneous narrowing of the mandibular angle, body, and chin while achieving a smooth and harmonious lower facial contour.

Compared with LC osteotomy combined with T-shaped genioplasty,^34^ this technique offers two major advantages. First, it is particularly suitable for patients with a low-positioned inferior alveolar nerve canal. Unlike T-shaped genioplasty, which requires removal of a horizontal bone fragment around or just below the mental foramen, the U-shaped osteotomy is performed well below the mental foramen. Second, the use of titanium screws or titanium plates is unnecessary.

Similar to the ABC osteotomy technique, the U osteotomy, as part of en-bloc MAO, requires extensive soft-tissue dissection and carries risks such as postoperative facial sagging and temporary lower-lip numbness. To address facial sagging, three holes were drilled along the mandibular margin for tension-free resuspension of the detached musculature. The semicircular free bone segment was divided into three smaller sections to facilitate removal and to minimize the risk of mental nerve injury. During long-term follow-up, they observed that patients who underwent the U osteotomy exhibited varying degrees of submental–cervical soft-tissue sagging, presenting a “double-chin” appearance. CT-based analyses further revealed that the increase in soft-tissue thickness was positively associated with the amount of osteotomy, and that the anterior belly of the digastric muscle showed a downward prolapse, contributing to the formation of the double chin.^35^ These findings highlight the importance of carefully considering the planned osteotomy volume during preoperative assessment.

#### Mandibular V-line osteotomy

In addition to curved osteotomy techniques, linear osteotomy procedures—exemplified by the Mandibular V-line osteotomy—have also been applied in current clinical practice. This technique was introduced by Hsu et al.^9^ as a treatment specifically designed for patients with a low gonial angle or those seeking an extremely slender oval facial contour. The osteotomy lines on both sides are symmetrically designed to create a configuration resembling the letter “V.” A low gonial angle is defined as a ∠Co–Go–Me of <110°—often around 90°—combined with an MP–HP angle of <20°, frequently approaching 0°.^24^^,^^36^ However, this technique is not suitable for patients with a high-positioned mandibular angle or an excessively short mandibular ramus, although no specific quantitative parameters have been clearly defined in the literature. Due to the use of a reciprocating saw, the procedure may be more accessible for less experienced surgeons, allowing for a smoother osteotomy line.

### Adjunctive procedures for mandibular contouring

The contour of the lower face is primarily determined by skeletal factors; therefore, it is shaped not only by the mandibular angle but also by the morphology of the mandibular body and chin and the masseter muscle. Consequently, comprehensive lower-face remodeling requires an integrated evaluation of multiple anatomical components. The following chapter discusses adjunctive procedures that can be performed simultaneously with MAO.

#### Mandibular outer cortex reduction techniques

An isolated MAO can improve the patient’s lateral profile, but it has limited effect on the frontal appearance. Therefore, Han et al.^4^ proposed a splitting corticectomy, which can effectively reduce the gonial-to-gonial distance. Zhang et al.^7^ subsequently introduced the mandibular outer cortex grinding technique and suggested that, compared with splitting corticectomy, this method offers several advantages: (1) it allows broader and safer access to otherwise difficult-to-expose surgical areas; (2) the ground bony surface is smoother than the surface obtained after splitting; (3) the appearance of cortical redness provides a timely indication that cancellous bone is about to be exposed, prompting cessation of bone removal; (4) the risk of injury to the inferior alveolar neurovascular bundle is lower during grinding; (5) it effectively reduces the likelihood of intraoperative fractures; and (6) it is more suitable for inexperienced or early-career surgeons.

#### Partial masseter muscle resection

Masseter muscle hypertrophy, which influences the lower facial contour, is a common concern among patients undergoing MAO.^37^ The masseter attaches to the mandible, and its contractile force plays a significant role in mandibular growth, particularly in the angle region.^38^ Consequently, some patients request simultaneous MAO and partial masseter muscle resection (PMMR).^10^, ^11^, ^12^ However, PMMR remains controversial, and it will be discussed in detail below.

#### Mandibular body osteotomy

The mandibular body serves as a transitional bridge between the mandibular angle and the chin and influences the oblique view. Hence, mandibular body osteotomy is indicated for patients who are dissatisfied with their oblique lower facial view. According to the mandibular tubercle resection technique described by Park et al.,^39^ the procedure is performed through a midline vestibular incision. A subperiosteal tunnel is created to access the parasymphyseal region, after which the tubercles are removed with a straight reciprocating saw and the contour refined with burring. Unlike narrowing genioplasty,^40^ this technique avoids step deformity and does not require fixation with plates and screws. In addition to Park’s report, Zhang et al.^41^ and Lee et al.^42^ have also described similar surgical techniques for correcting the lower border of the mandible, referred to as the oblique mandibular chin-body osteotomy and the mini V-line osteotomy, respectively.

#### Surgical correction techniques for chin deformities

Patients with specific chin deformities, such as microgenia or retrogenia, often require additional corrective procedures, including implant-based augmentation and osseous genioplasty (OG). In patients with mild-to-moderate chin deficiencies—most commonly involving sagittal insufficiency at the pogonion or transverse deficiencies at the symphysis—implant-based chin augmentation is associated with less postoperative discomfort and faster recovery, as reported in previous studies.^43^^,^^44^ For severe microgenia, a combined approach may be beneficial; Findikcioglu et al.^45^ demonstrated successful outcomes using OG together with Medpor augmentation in three such cases. Kauke-Navarro et al.^46^ reported higher satisfaction trends with OG, along with distinct complication profiles: implants had greater infection risk, whereas OG showed more neurosensory disturbances. Relapse was slightly higher with OG, which nonetheless offered superior soft-tissue predictability (85%vs 66%). Despite these findings, systematic treatment algorithms for chin augmentation remain underdeveloped, underscoring the need for future research to refine indications and optimize procedural selection.

### Controversies in mandibular angle osteotomy

#### Curved osteotomy vs. linear osteotomy

Controversy remains regarding curved osteotomy^3^^,^ ^7^^,^ ^8^ versus linear osteotomy.^9^^,^^36^ These techniques differ in both remodeling objectives and postoperative contour characteristics. A comparison of the two techniques is presented in Table 2. Curved osteotomy—characterized by a long, continuous arc extending from the mandibular ramus to the inferior border—more closely conforms to the native mandibular curvature and has been widely adopted to achieve a smoother and more continuous mandibular border.^3^^,^^7^^,^^8^^,^^47^ In contrast, linear osteotomy primarily reshapes the mandibular angle using a relatively straight osteotomy line^9^^,^^36^; although technically straightforward, it has been associated with postoperative contour irregularities, such as step deformities.^48^

**Table 2:** Comparison of the advantages and disadvantages between linear and curved mandibular angle osteotomy techniques.

Osteotomy type	Pros	Cons
Curved osteotomy	• Better conformity to native mandibular curvature	• Technically demanding with a steeper learning curve
	• Smoother and more continuous postoperative mandibular border	• Higher risk of cutting deviation and unintended ramus fracture
		• Potential loss of ramus height
		• Possible increased risk of soft-tissue ptosis
Linear osteotomy	• Technically simpler and more reproducible	• May result in contour irregularities (e.g., step deformities)
	• Better preservation of ramus height	
		• Limited reshaping of the mandibular border
		• Oblique osteotomy plane due to restricted working space

Regarding the surgical instruments used in these two osteotomy techniques, each presents distinct technical challenges. Curved osteotomy is usually performed with an oscillating saw. However, the arc-shaped cutting path increases the chance of deviation and unintended ramus fractures—an outcome strongly influenced by the surgeon’s experience.^3^^,^^7^^,^^8^ Linear osteotomy, performed with a reciprocating saw to create a straight-line osteotomy at the mandibular angle, is constrained by the limited space perpendicular to the mandibular surface. This restriction often results in an oblique osteotomy plane between the inner and outer cortices.^9^^,^^36^

In a comparative study by Han et al.^49^ evaluating V-line osteotomy and long curved osteotomy, no significant differences were observed in the postoperative mandibular angle (116.67 ± 7.14° vs. 118.31 ± 6.80°, *P* = 0.233) or symmetry indices (2.36 ± 1.21 mm vs. 2.56 ± 1.19 mm, *P* = 0.395). However, patients undergoing V-line osteotomy demonstrated a significantly greater postoperative ramus height and a smaller curvature of the osteotomy line (*P* < 0.001). The authors suggested that loss of ramus height may compromise the physiological contour of the mandibular angle and reduce structural support for overlying soft tissues, potentially increasing the risk of soft-tissue ptosis. Nonetheless, quantitative studies comparing soft-tissue changes following these two osteotomy patterns remain lacking. Facial sagging may be mitigated by drilling three holes along the mandibular margin to allow tension-free resuspension of the detached musculature^8^ or by periosteal suturing.^6^ In addition, adjunctive procedures—such as rhytidectomy, botulinum toxin type A injections, or radiofrequency treatments—may further improve aesthetic outcomes in selected cases undergoing curved osteotomy.^50^, ^51^, ^52^, ^53^

Taken together, these findings suggest that neither curved nor linear osteotomy is universally superior in terms of objective skeletal outcomes. Instead, the choice of osteotomy design should be guided by individual mandibular morphology, aesthetic priorities, and, importantly, the surgeon’s technical experience. Given the steeper learning curve and higher technical demands of curved osteotomy, surgeon familiarity and proficiency may play a critical role in minimizing complications and optimizing aesthetic outcomes.

#### Should partial masseter muscle resection be performed?

PMMR was first introduced by Gurney in 1947,^54^ predating the initial reports of MAO. Whether PMMR should be performed simultaneously with MAO remains a subject of ongoing debate. Although numerous studies have measured and analyzed postoperative changes in masseter volume, morphology, and function following MAO, the findings to date remain inconclusive.

Authors who oppose simultaneous PMMR noted that the masseter muscle tends to exhibit postoperative atrophy after MAO, and the atrophy rate varies substantially among individuals. First, detachment of the masseter muscle from mandible leads to atrophy.^55^ This mechanism is analogous to the muscle changes observed following tendon rupture.^56^ At the same time, the masseter has not yet re-established its attachment to the mandible, resulting in reduced muscle activity and further exacerbation of atrophy. Second, detachment disrupts the supportive structural unit formed by the vertical alignment of the masseter’s origin and insertion.^57^ In the long-term follow-up, the masseter muscle volume decreased by 20.98% to 39.58% according to CT data.^58^, ^59^, ^60^

Scholars who oppose concomitant masseter resection also argue that the procedure carries a risk of vascular and neural injury and may even compromise occlusion function.^61^^,^^62^ The masseteric nerve arises from the mandibular nerve trunk and courses anteroinferiorly between the middle and deep layers, giving branches to the entire muscle. Superior and inferior posterior branches innervate the deep and middle layers, respectively,^63^ and small intramuscular branches form multiple anastomoses.^11^ This neural distribution implies that deep resection of the middle or lower masseter carries a significant risk of nerve injury.

Some authors have suggested that resection of the inner layer of the masseter muscle is unlikely to injure the masseteric artery, as the artery is primarily located in the superficial layer and courses inferiorly and anteriorly toward the middle portion of the muscle.^64^ However, it is important to note that a deep middle masseteric artery, originating from the external carotid or transverse facial artery, enters the posterior aspect of the masseter muscle 31 mm below the mandibular angle and runs along its deep surface.^65^

According to a comparative study evaluating patients who underwent MAO with or without PMMR,^10^ the MAO+PMMR group demonstrated a significantly greater reduction in masseter volume (*P* < 0.001) and a greater decrease in facial width (*P* < 0.001), along with less bone regeneration (*P* < 0.001). Moreover, patient-reported satisfaction was higher in the MAO+PMMR group (*P* < 0.05). The authors suggested that concomitant masseter resection markedly reduces masseter attachment at the mandibular angle, thereby decreasing mechanical stimulation and subsequent osseous proliferation. In addition, periosteal removal during deep-layer resection may further contribute to the reduced bone regeneration observed.

Whether PMMR should be performed concurrently depends on the degree of masseter hypertrophy, and requires a comprehensive assessment of both hard and soft tissues. For patients with mild masseter hypertrophy, isolated MAO is generally sufficient, whereas those with severe hypertrophic masseter muscles may benefit from simultaneous PMMR.

### Clinical scenario-based decision-making

To improve the practical applicability of this review, representative clinical scenarios are summarized to illustrate how mandibular morphology, aesthetic goals, technical difficulty, and surgeon experience may guide surgical planning (Table 3). For patients with an extroverted mandibular angle and adequate chin projection, long curved osteotomy combined with mandibular outer cortex reduction may provide sufficient improvement in both lateral and frontal views. For patients with a low gonial angle who desire a narrower lower face, V-line osteotomy may be considered, although excessive reduction of ramus height should be avoided to preserve the physiological mandibular contour and soft-tissue support. For patients with a broad or long chin, isolated MAO is usually insufficient; ABC osteotomy, U-shaped osteotomy, or genioplasty-based combined procedures may be required. In patients with severe masseter hypertrophy, partial masseter muscle resection may be considered after careful assessment of the risks of neuromuscular injury, functional impairment, and postoperative atrophy. Digital osteotomy templates or navigation-assisted techniques may be particularly useful in patients with introverted mandibular angles, asymmetry, or limited intraoral exposure.

**Table 3:** Clinical scenario-based surgical recommendations.

Clinical scenario	Main concern	Suggested strategy	Key caution
Introverted mandibular angle	Limited exposure and difficult access	DOT-assisted MAO ± MOCR	Avoid osteotomy deviation
Broad chin with PMA	Wide lower third	ABC osteotomy, U-shaped osteotomy, or LC + MBO	Protect the mental nerve
Broad chin with PMA	Wide lower third	ABC osteotomy, U-shaped osteotomy, or LC + MBO	Protect the mental nerve
Recessive chin	Insufficient chin projection	LC + advancing genioplasty	Maintain chin–mandible harmony
Wide lower face after osteotomy planning	Excessive frontal width	Additional MOCR	Avoid over-reduction
Inexperienced surgeon	Technical difficulty and safety	DOT, segmented osteotomy, or outer cortex grinding	Reduce risk of fracture and nerve injury

### When are emerging technologies needed in mandibular angle osteotomy?

Although emerging technologies such as virtual surgical planning, digital osteotomy templates, dynamic navigation, and endoscopic assistance have expanded the technical options for mandibular angle osteotomy, their routine use in all patients may not be necessary. The clinical value of these technologies should be considered according to mandibular morphology, surgical complexity, the need for high accuracy, intraoperative exposure, cost-effectiveness, and surgeon experience. Rather than being regarded as mandatory tools, these technologies should be selectively applied to improve safety, accuracy, and reproducibility in anatomically complex or technically demanding cases.

**Figure 5:**
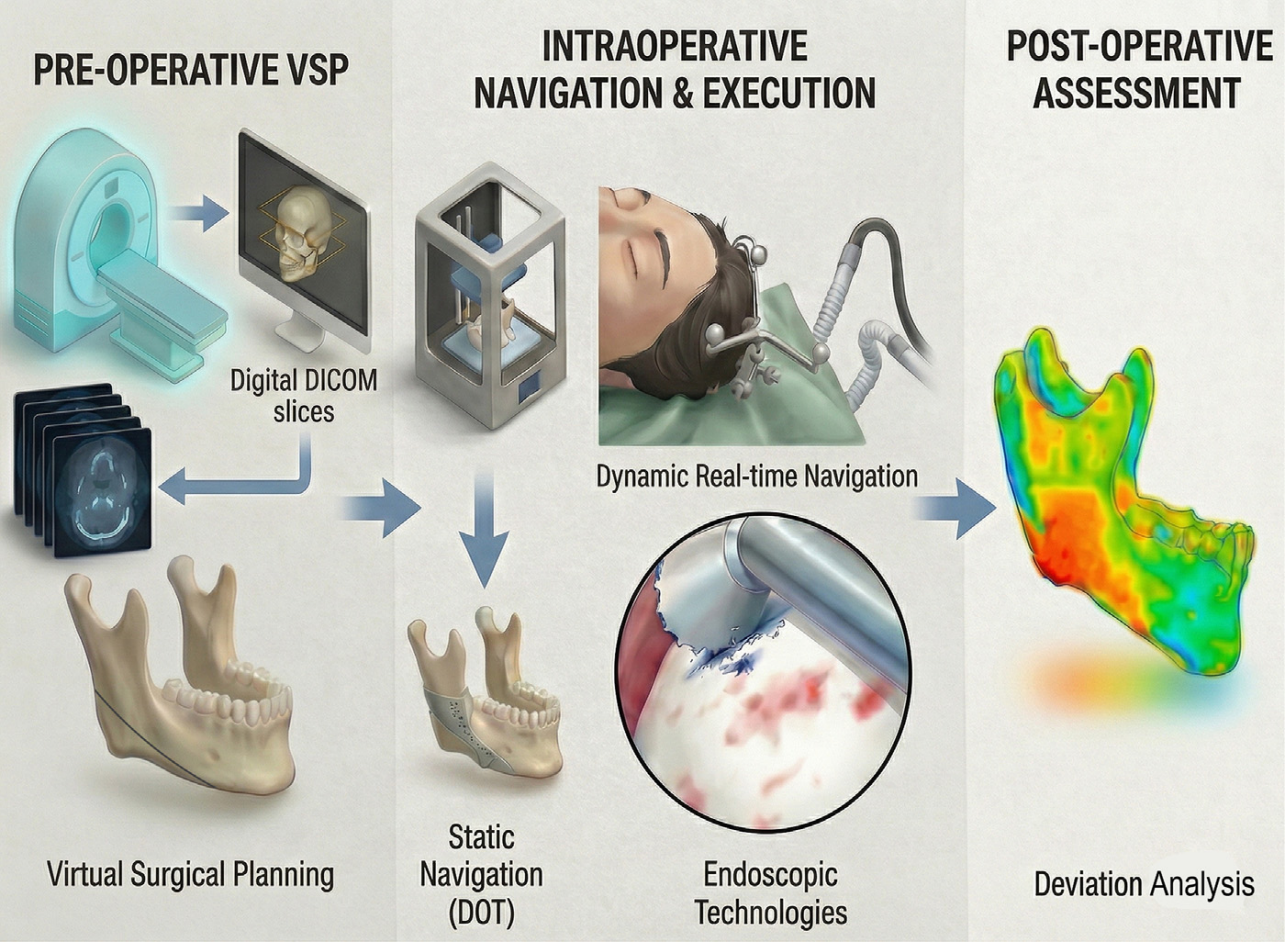
Workflow of novel technologies applied in mandibular angle osteotomy.

In general, emerging technologies may be particularly useful in the following situations: patients with introverted mandibular angles, severe asymmetry, low or atypical position of the inferior alveolar nerve canal, limited intraoral exposure, complex combined procedures involving the mandibular angle, body, and chin, or cases in which accurate reproduction of the preoperative plan is critical. They may also help less-experienced surgeons better understand the three-dimensional anatomy and reduce the learning curve. Conversely, in patients with relatively straightforward mandibular morphology and in procedures performed by experienced surgeons, conventional osteotomy techniques may remain sufficient. Figure 5 illustrates the workflow and potential clinical applications of these technologies.

#### Virtual surgical planning: defining surgical targets and evaluating outcomes

The general procedure for virtual surgical planning (VSP) and postoperative outcome assessment is as follows. Raw medical imaging data obtained from computed tomography (CT) or magnetic resonance imaging (MRI) in Digital Imaging and Communications in Medicine (DICOM) format are imported into dedicated software platforms, such as MIMICS, ProPlan CMF, Blender, and 3-matic, to reconstruct a three-dimensional (3D) virtual model of the craniofacial skeleton. Smoothing procedures to remove imaging artifacts, as well as the addition of supporting structures when necessary, are subsequently performed to further refine and enhance the accuracy of the segmented anatomical structures.^66^ The generated three-dimensional model displays the trajectory and relative positioning of the mandibular nerve canal in the coronal, sagittal, and axial planes. Based on clinical measurements and patients’ requirements, the osteotomy lines are designed to execute the VSP.^67^

Simon et al.^14^ developed a facial gender-affirming surgery planning software co-developed with the Nemotec SL platform to perform VSP for 837 patients scheduled to undergo facial gender-affirming procedures. The surgical procedures included MAO and genioplasty. Ladnier et al.^13^ reported the case of a young woman with familial mandibular fibrous dysplasia who presented with progressive bilateral mandibular swelling. Before surgery, VSP was performed using her cone-beam computed tomography (CBCT) data, which were superimposed onto an age-matched standard mandibular model to design and fabricate a repositioning guide to direct intraoperative osteotomies. At the 18-month postoperative follow-up, no further mandibular expansion was observed, and the surgically remodeled mandible remained stable.

The postoperative assessment protocol was identical to that used for VSP. Zhao et al. ^68^and Choi et al.^69^ extracted three-dimensional mandibular models to measure and evaluate volumetric changes of the mandible preoperatively, in the short-term postoperative period, and in the long-term postoperative period based on MIMICS software and CT data. Several studies have identified changes in mandibular morphology associated with the masseter muscle,^70^ immune factors,^71^ bone density,^72^ and the levels of red blood cells and hemoglobin.^26^ In addition to longitudinal follow-up and monitoring of dynamic changes in hard and soft tissues after surgery, many investigators have employed three-dimensional image superimposition techniques, matching VSP-generated images with actual postoperative images to assess surgical accuracy.^13^^,^^73^ This approach enables the identification of sites of surgical error, assists surgeons in analyzing the underlying causes of these discrepancies, and allows intuitive visualization of dynamic changes in the mandible, masseter muscle, and other related structures, thereby providing guidance for optimizing the extent of osteotomy in future procedures.

#### Digital osteotomy templates: when static guidance is helpful

The limited intraoral surgical field and restricted operating space are major obstacles that may contribute to suboptimal contouring and serious complications. Contemporary navigation systems can be broadly classified into static systems using patient-specific osteotomy templates and dynamic navigation systems based on computer-assisted technologies.

The design and fabrication of three-dimensional printed Digital Osteotomy Template (DOT) follow the same workflow as virtual surgical planning. First, CT data in DICOM format are imported into the surgical planning software and converted into three-dimensional reconstructed images.^20^ The transparency of the hard and soft tissues in the three-dimensional model is adjusted to visualize critical anatomical structures, including the mandible and the inferior alveolar nerve. Osteotomy lines are then designed to meet the patient’s aesthetic expectations while ensuring safety. After completion of the VSP, the design data are saved in STL format and imported into reverse-engineering software such as GeoMagic to generate a digital model. Digital osteotomy templates are created using Boolean operations, whereby the virtually planned postoperative mandibular model is subtracted from the original mandibular model.^16^ Finally, the templates are fabricated with a 3D printer using materials such as polylactic acid, polyacrylic acid, or photosensitive resin, and the edges are polished to obtain a smoother surface and facilitate placement in the surgical field.^21^

Rong et al.^16^ first applied a stereolithographically fabricated surgical guide to bony resection in unilateral PMA in 2013. Subsequent clinical applications have further confirmed the safety and efficacy of digital osteotomy templates.^7^^,^^18^^,^^20^^,^^22^ However, these DOTs are typically designed based on the resected mandibular segment, which can make placement challenging, particularly in cases of posteriorly protruding PMA. To address this issue, Wu et al.^17^ developed a novel DOT featuring three hooks positioned at the posterior border, anterior ramus, and inferior border of the mandible, and reported a reduced operative time. AI-based DOTs have been preliminarily applied in MAO, reducing preoperative design time and improving postoperative facial symmetry.^15^^,^^19^ For patients with an introverted mandibular angle, DOT is recommended, as the limited intraoral surgical access makes it difficult to adequately expose the morphology and spatial position of the mandibular angle. In addition, DOT is also recommended for patients with bilateral asymmetric mandibular angles, as it allows for maximal reproduction of the preoperative VSP.

At present, the application of AI in cranio-maxillofacial surgery remains at an early stage. Future advances may include the establishment of dedicated databases for MAO to support surgical planning and open new research directions. Looking ahead, research on DOTs is expected to focus on improving surgical accuracy, enhancing fixation and intraoperative stability, and optimizing the biocompatibility of template materials.

#### Dynamic real-time navigation: when continuous intraoperative guidance is needed

In MAO, dynamic real-time navigation has emerged as a complementary strategy to static digital osteotomy templates, enabling continuous guidance of osteotomy orientation and depth during osteotomy. Current navigation solutions used or explored in MAO can be broadly categorized by the tracking modality: (1) optical tracking–based systems, which track the mandible and instruments via infrared cameras and reflective/active markers^74^; (2) electromagnetic tracking–based systems, which provide line-of-sight–independent tracking through field generators and sensors^75^; (3) hybrid optical–electromagnetic tracking–based systems, which combine optical tracking (often for instruments) with electromagnetic tracking (often for the mandible) to mitigate line-of-sight occlusion and improve robustness in confined operative fields and^76^; (4) navigation-enhanced visualization systems, such as augmented reality (AR) overlays that present planned osteotomy planes and safety margins intraoperatively, typically relying on optical/vision-based registration.^77^ In some settings, navigation may also be integrated with robot-assisted workflows to improve trajectory consistency.^75^

For MAO specifically, dynamic navigation is particularly valuable because mandibular motion and limited posterior surgical access can compromise the placement and stability of static guides. By fixing a reference frame to the mandible (or dentition) and registering the preoperative plan to the intraoperative anatomy, navigation provides real-time feedback on the position of the saw/osteotome relative to the planned osteotomy line and critical structures, thereby supporting postoperative symmetry and reducing the risk of malpositioned osteotomy. Nevertheless, its performance remains sensitive to registration accuracy and intraoperative stability of the reference frame; optical systems may suffer from line-of-sight interruption in posterior regions, whereas electromagnetic systems may be affected by metallic interference. Moreover, this technique is still at a preliminary experimental stage, has not yet been widely applied, and remains costly.

Therefore, future work in MAO is expected to focus on improving robust mandibular registration, mitigating motion-related errors, and integrating navigation with personalized planning to enhance reliability and reproducibility. If these challenges are addressed, this technology may become an excellent tool for young surgeons and help reduce their learning curve.

*Endoscopic Assistance: When Direct Visualization Is Limited*In plastic surgery, endoscopic techniques have been successfully applied to procedures such as breast augmentation and rhytidectomy.^78^ In cranio-maxillofacial surgery, endoscopy has also been widely used for facial bone fracture reconstruction.^79^^,^^80^ Endoscope-assisted MAO via an intraoral approach has become a well-established technique.^32^ By expanding the operative field, endoscopy enables more accurate identification of anatomical structures that are difficult to expose under direct vision, particularly in the mandibular angle and ramus regions. This advantage is especially valuable in patients with severe mandibular angle inset or facial asymmetry, in whom the osteotomy line can be determined more reliably, thereby minimizing osteotomy deviation. In addition, endoscopic assistance facilitates precise and safe intraoral masseter reduction when indicated.^81^ Endoscope-assisted surgery has also been associated with reduced intraoperative blood loss and a lower risk of complications, including unintended mandibular fractures and facial nerve injury.

Despite providing a sufficiently clear surgical view, endoscopy converts a conventionally three-dimensional operative task into a two-dimensional display. This change imposes a learning curve and typically requires structured, long-term training to achieve proficiency.

### Long-term mandibular remodeling and morphological changes

According to Yu et al.,^82^ undercorrection of the mandibular angle is the leading reason for patients undergoing revision mandibular angle surgery, accounting for 88% of admissions. Even when the operation is completed as planned and yields a satisfactory immediate postoperative appearance, long-term mandibular morphology may still change due to postoperative remodeling.

Using CT-based three-dimensional reconstruction, Zhao et al.^83^ evaluated outcomes at 6 months after resection of the mandibular lateral cortex and quantified variables related to bone regeneration. They reported evident new bone formation at the resected sites, with a regeneration rate of up to 84.6%. Lo et al.,^60^ in a long-term follow-up of seven patients undergoing mandibular angle contouring osteotomy, observed cortical thickening and partial regeneration in the angle region. In addition, Zhao et al.^68^ found that postoperative morphological changes involve not only bone regeneration but also varying degrees of bone resorption. Gu et al.^71^ identified factors associated with the bone regeneration rate, showing a positive association with preoperative monocyte count and negative associations with age. In addition, Xie et al.^26^ and Gu et al.^72^ reported that remodeling in the mandibular angle region correlates with red blood cell count, hemoglobin level, and CT-derived bone density.

Such remodeling may contribute to recurrent PMA, an irregular mandibular inferior border line, and postoperative facial asymmetry. Therefore, a better understanding of long-term mandibular remodeling after MAO may provide more reliable guidance for surgical planning and follow-up, thereby minimizing the likelihood of secondary revision procedures.

### Limitations and future directions

This review is limited by the heterogeneity and overall low evidence level of the available literature, which is predominantly composed of retrospective case series. Variations in patient selection, osteotomy design, adjunctive procedures, outcome measures, and follow-up duration hinder direct comparison among MAO techniques. In addition, quantitative data on long-term soft-tissue changes and patient-reported outcomes remain limited. Although emerging digital and navigation-assisted technologies show promise, their cost-effectiveness, learning-curve impact, and generalizability have not been fully established. Future studies should emphasize prospective designs with standardized morphological classifications, objective three-dimensional assessments, and long-term follow-up to better define optimal surgical strategies and support personalized MAO planning.

## Conclusion

Mandibular angle osteotomy in Asian patients should be regarded as an individualized lower-face contouring procedure rather than a simple angular reduction operation. Surgical planning should integrate mandibular morphology, chin proportion, masseter volume, soft-tissue characteristics, patient expectations, and surgeon experience. Curved osteotomy may provide a smoother mandibular contour but is technically more demanding, whereas linear or V-line osteotomy may be useful in selected patients with a low gonial angle. Adjunctive procedures, including mandibular outer cortex reduction, genioplasty, mandibular body osteotomy, and partial masseter muscle resection, should be selected according to clearly defined surgical endpoints. Emerging digital, endoscopic, and navigation-assisted technologies may improve surgical accuracy and safety, particularly in anatomically complex cases. Future studies should focus on standardized outcome assessment, patient-reported satisfaction, and long-term three-dimensional evaluation of both skeletal and soft-tissue remodeling.

## Declaration

### Ethical approval

This article does not contain any studies with human participants or animals performed by any of the authors.

### Informed consent

For this type of study, informed consent is not required.

## Funding

None.

## Declaration of competing interest

The authors declare that they have no conflict of interest.
